# Imaging Features Following Breast Explant Surgery: A Pictorial Essay

**DOI:** 10.3390/diagnostics13132173

**Published:** 2023-06-26

**Authors:** Yusuf T. Akpolat, Mark J. Dryden, Marion E. Scoggins, Miral M. Patel, Ceren Yalniz, Victor J. Hassid, Gary J. Whitman

**Affiliations:** 1 Department of Breast Imaging, The University of Texas MD Anderson Cancer Center, Houston, TX 77030, USA; ytakmd@gmail.com (Y.T.A.); mdryden@mdanderson.org (M.J.D.); marimscoggins@gmail.com (M.E.S.); mpatel6@mdanderson.org (M.M.P.); 2 Department of Radiology Breast Imaging Section, The University of Alabama at Birmingham, Birmingham, AL 35233, USA; cerenyalniz@gmail.com; 3 Department of Plastic Surgery, The University of Texas MD Anderson Cancer Center, Houston, TX 77030, USA; vjhassid@mdanderson.org

**Keywords:** breast implant removal, breast explantation surgery, breast implant illness, imaging findings, explantation findings, capsular contracture, implant rupture

## Abstract

Breast implants can be removed with breast explantation surgery (BES) for various reasons, including patient dissatisfaction, capsular contracture, implant infection or rupture, breast implant-associated anaplastic large cell lymphoma, and a recently emerging phenomenon called breast implant illness. There is very limited data on the imaging appearance after BES. A retrospective chart review was performed for patients with BES findings on imaging reports for the period between October 2016 and October 2021. When assessing BES techniques, a key element is determining whether the implant’s fibrous capsule requires removal. The second important question is if the patient requires an additional aesthetic procedure after BES. BES techniques include capsulotomy, and partial, total, or en bloc capsulectomy. Adjunctive aesthetic or reconstructive procedures after BES include fat grafting, mastopexy, augmentation, and reconstruction with flaps. The majority of post-BES breast imaging findings are related to the surgical scar/bed, thereby confirming that the type of explantation surgery is important. Imaging findings after BES include focal and global asymmetries, architectural distortions, calcifications, calcified and non-calcified fat necrosis, masses, hematomas, seromas, capsular calcifications, and silicone granulomas. Most importantly, since these patients have residual breast tissue, paying attention to imaging features that are suspicious for breast cancer is necessary.

## 1. Introduction

Breast implant procedures are commonly performed as cosmetic surgical procedures [1]. Breast implants may also be placed for breast reconstruction following mastectomy in women with breast cancer. Like every surgical procedure, breast implant placement has side effects and complications. The most common complications of breast implants are capsular contracture, implant rupture, breast implant-associated anaplastic large cell lymphoma (BIA-ALCL), and a recently emerging phenomenon in the literature called breast implant illness [2,3,4,5,6,7,8]. Breast implants can be removed with or without reimplantation with breast explantation surgery (BES).

In the era of radiologists being consultant physicians, it is the breast imagers’ responsibility to be informed about BES in order to differentiate between benign post-surgical changes and findings suspicious for breast cancer. The imaging appearances after breast implant-based reconstruction and breast augmentation surgeries are well described in the literature [9,10,11,12,13]. In contrast, there is very limited data on the imaging appearance after BES. Hayes et al. described the mammographic findings after the removal of breast implants in 1993 [14]. In 1995, Soo et al. described seromas in residual fibrotic capsules on mammography and ultrasound after BES [15]. No papers were found in the literature with a systematic approach to the imaging findings after BES, including mammography, ultrasound, and magnetic resonance imaging (MRI). In the ideal situation, comparison of images before implantation surgery, while the implant is in place, and after BES would be helpful for the radiologist. Often pre-implant, implant, and prior explant images are not available, and it is important for the radiologist to recognize typical BES findings in order to prevent unnecessary follow-ups or biopsies. In summary, despite increasing data and awareness on breast implant-related complications that may require BES, there is limited data on the imaging findings after BES. In this paper, we discuss BES techniques and describe the imaging findings following BES.

### Breast Explantation Surgery Techniques

There are different BES techniques according to patients’ preferences and needs. The most important question to ask in BES is the removal of the fibrous implant capsule. The second important question is if the patients require or want additional procedures following explantation. These surgeries include capsulotomy, partial vs. total vs. en bloc capsulectomy, and BES with adjunctive aesthetic or reconstructive procedures, including fat grafting, mastopexy, and reconstruction with flaps [16,17,18].

Capsulotomy involves an incision through the periprosthetic capsule. Partial or anterior capsulectomy involves excising a portion of the capsule, mostly the anterior capsule, and leaving a portion of the capsule, usually the posterior aspect, intact. The total capsulectomy procedure removes the entire capsule.

En-bloc resection is often a misused term that means cancer removal in a BIA-ALCL diagnosed patient with removal of the implant, complete capsule in conjunction with any associated mass and a rim or margin of surrounding healthy tissue [19].

**Figure 1:**
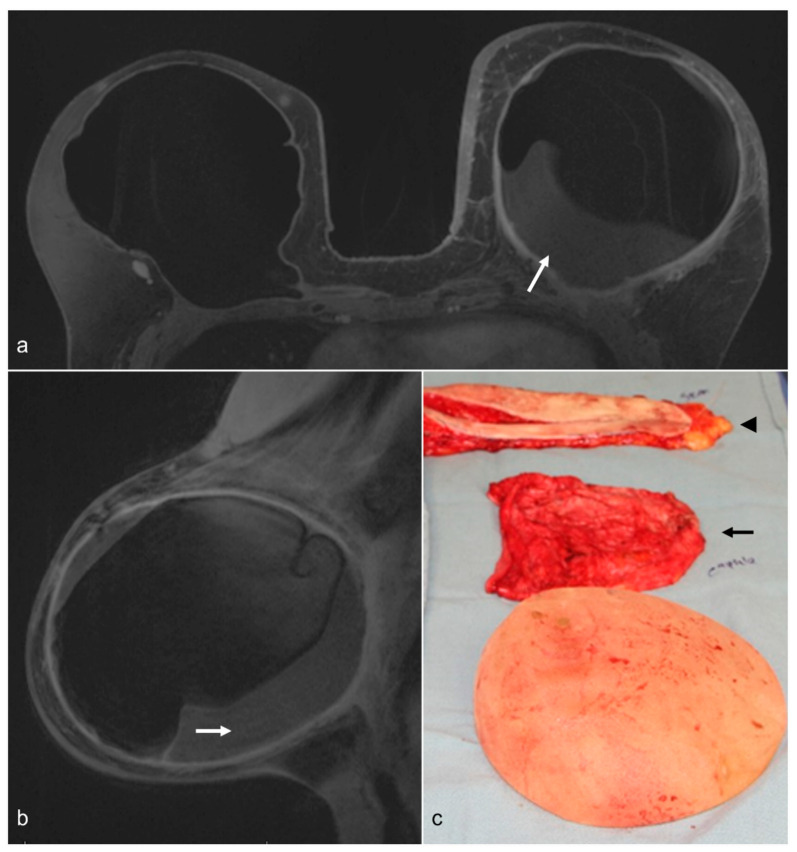
A 58-year-old woman with a history of bilateral prophylactic mastectomy and implant reconstruction. The patient had capsular contracture and a chronic fluid collection in the left breast (white arrows) on T1-weighted contrast-enhanced axial (**a**) and left sagittal (**b**) images. She underwent BES, and a photograph of the surgical specimen (**c**) shows the removed implant, the fibrous capsule (black arrow), and skin (arrowhead).

Capsulectomy is usually performed through existing incision scars, which have been used for cosmetic or reconstructive purposes. Surgeons at times need to extend those incisions in order to optimize visualization and safely perform removal of the portion or the entire capsule (Figure 1). For patients with retropectoral implants, if total capsulectomy is performed, surgeons need to pay particular attention to the posterior layer of the capsule, which is often adherent to the anterior chest wall, in order to avoid iatrogenic pneumothorax.

The decision regarding removing the capsule mostly depends on the underlying diagnosis, including BIA-ALCL, the degree of capsular contracture, patient goals and expectations, surgical planning, and intraoperative findings. Silicone implant rupture with a thickened capsule and/or silicone embedded into the capsule may also require capsulectomy.

After BES, the remainder of the breast mound is often addressed with breast contouring and/or volume restoration techniques involving mastopexy, fat grafting, augmentation with flaps, or new implants.

## 2. Findings

Imaging features after explantation surgery vary depending on the cause of the explantation, the BES technique, and the time since the explantation surgery. Radiologists working mostly with aesthetic surgeons may see post-explantation findings, and radiologists working at large referral cancer centers might see post-explantation findings along with findings due to breast conservation therapy and/or oncoplastic rearrangement.

The majority of the findings are related to the surgical scar/bed, thus identifying that the prior explantation surgery is important if it is not given as a history. Findings include focal and global asymmetries, architectural distortions, breast calcifications, calcified and non-calcified fat necrosis, masses, hematomas, seromas, capsular calcification, and silicone granulomas. The severity of the above findings is usually proportional to the time elapsed since BES. Most importantly, since these patients have residual breast tissue, paying attention to features suspicious for breast cancer is necessary.

### 2.1. Global Asymmetry

**Figure 2:**
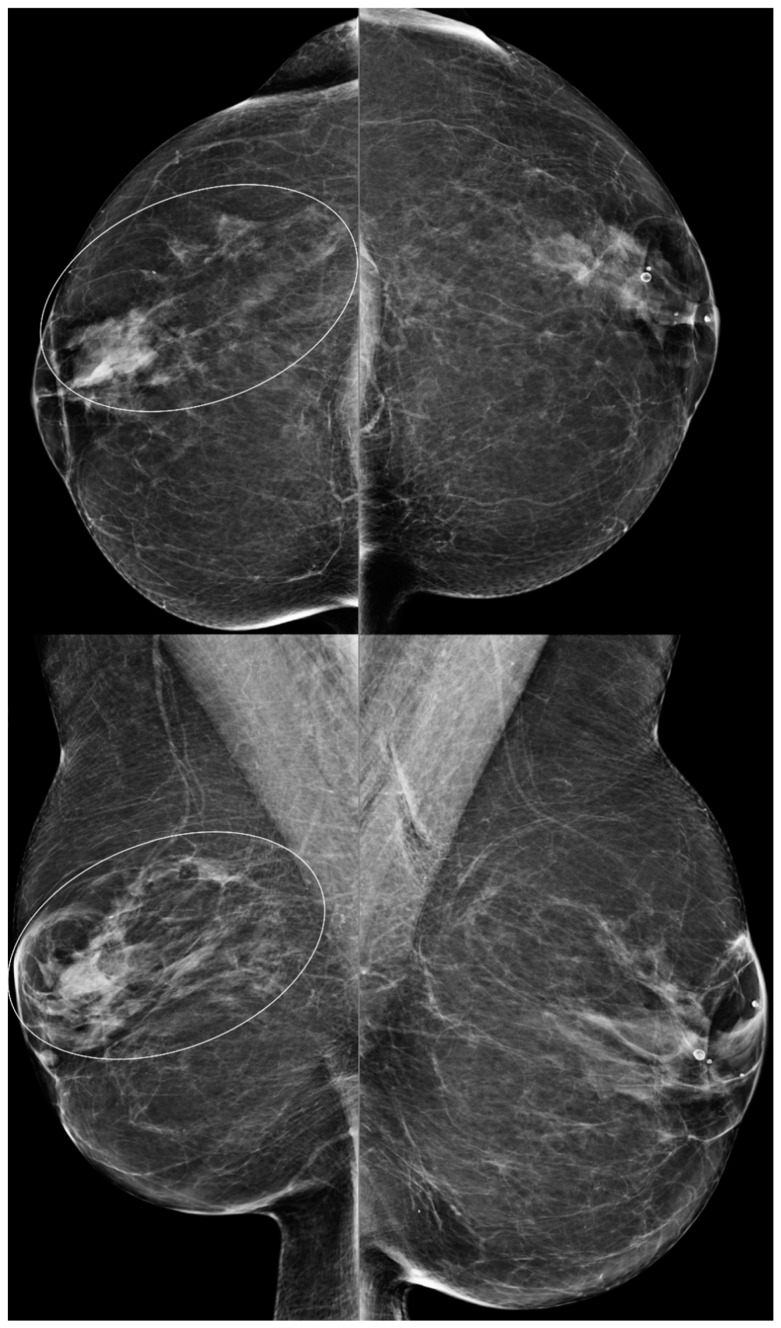
A 79-year-old woman with BES changes. Bilateral CC (**above**) and MLO (**below**) views demonstrate more dense tissue from post-BES changes on the right (ovals) compared to left breast. Note that right breast is smaller than the left breast.

Global asymmetry is described as a large amount of fibroglandular density tissue over a substantial portion of the breast and judged relative to the corresponding area in the contralateral breast. BES can cause global asymmetry in the bilateral breasts due to differences in the granulation response of the breast tissue after surgery (Figure 2).

### 2.2. Architectural Distortion

**Figure 3:**
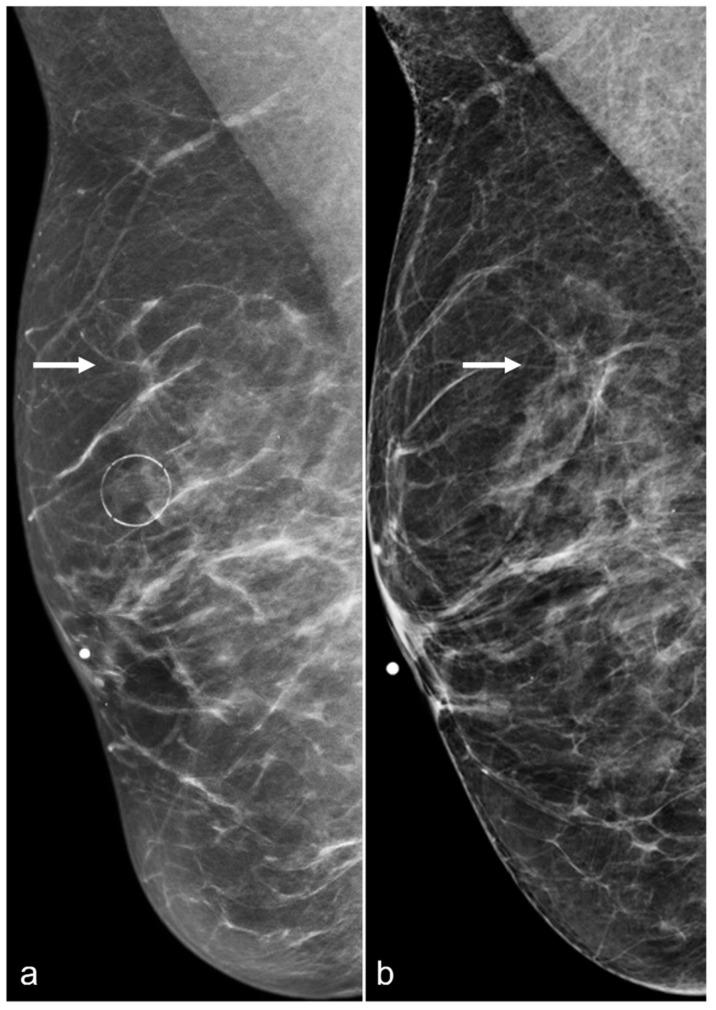
A 61-year-old woman had a history of subglandular silicone implants, status post BES. Surgical changes including architectural distortion (arrows) were noted in the right breast, stable for two years. ((**a**) Synthetic 2D MLO view from 2019; (**b**) 2D MLO view from 2021). The findings were consistent with post-BES change.

Architectural distortions can be seen in BES patients due to focal areas of fibrosis. Since architectural distortion is a suspicious finding, further evaluation with spot compression images or tomosynthesis is usually suggested (Figure 3).

### 2.3. Calcifications/Fat Necrosis

**Figure 4:**
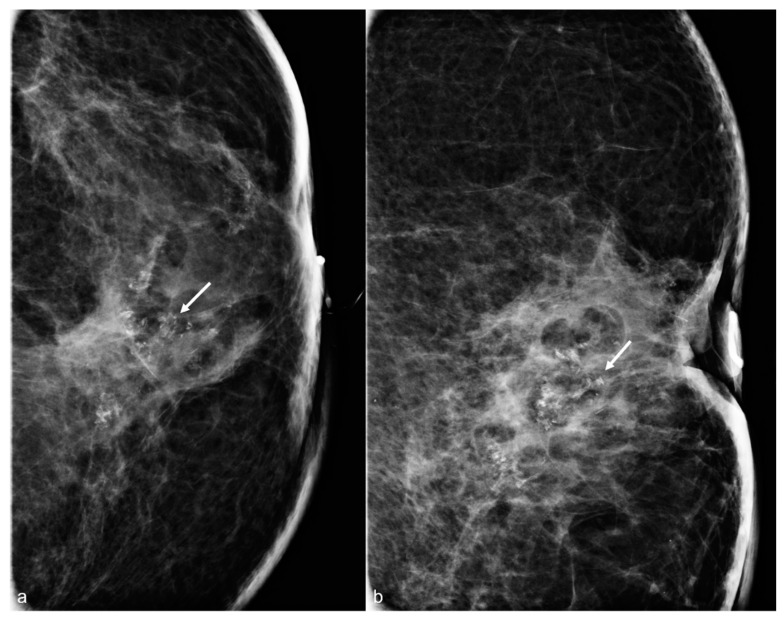
A 65-year-old woman with a history of subglandular silicone implants. Mammograms with magnified CC (**a**) and ML (**b**) views demonstrate calcified (arrows) and non-calcified fat necrosis in the left central breast. The patient underwent BES four years earlier.

**Figure 5:**
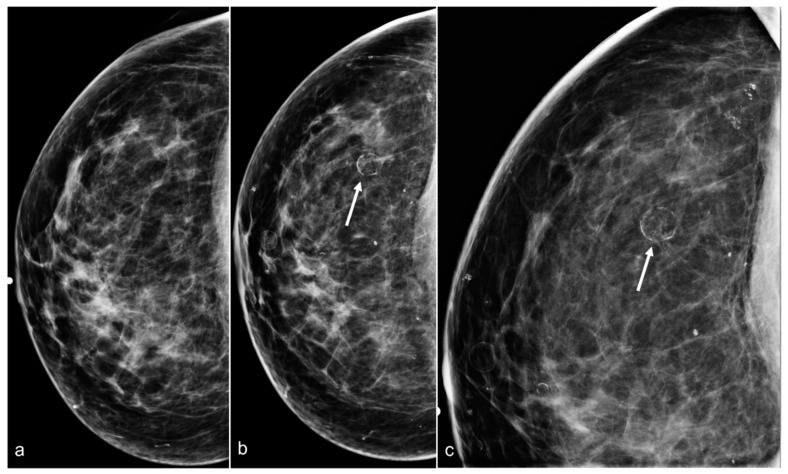
A 54-year-old woman with a history of subglandular silicone implants. Right breast CC view (**a**) performed 2 years after BES and mammogram performed 3 years after BES CC (**b**) and magnified CC (**c**) views demonstrate developing oil cysts (arrows) in the right breast.

**Figure 6:**
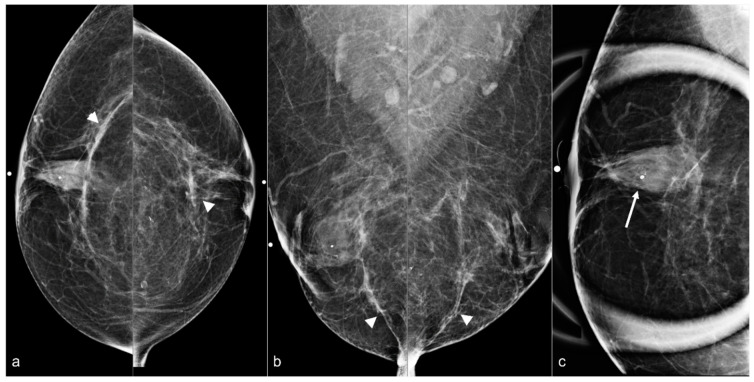
A 58-year-old woman with a history of subglandular silicone implants. BES was performed 1 year before the current mammogram. Bilateral CC (**a**) and MLO views (**b**) demonstrate bilateral linear densities indicating fibrous capsules (arrowheads) and bilateral, right greater than left, retroareolar focal asymmetries related to BES. Grouped amorphous calcifications (arrow) seen on a magnified CC view (**c**) in the right breast retroareolar region were biopsied with stereotactic guidance, with pathology demonstrating fat necrosis and giant cell reaction.

BES often leads to the development of fat necrosis, noted as oil cysts and dystrophic calcifications. The extent of the fat necrosis calcifications varies from patient to patient. Some patients have subtle calcifications (Figure 4), and some have more pronounced calcifications with oil cysts (Figure 5), or some even have suspicious calcifications (Figure 6).

### 2.4. Asymmetries and Masses Anterior to the Pectoralis Major Muscle

**Figure 7:**
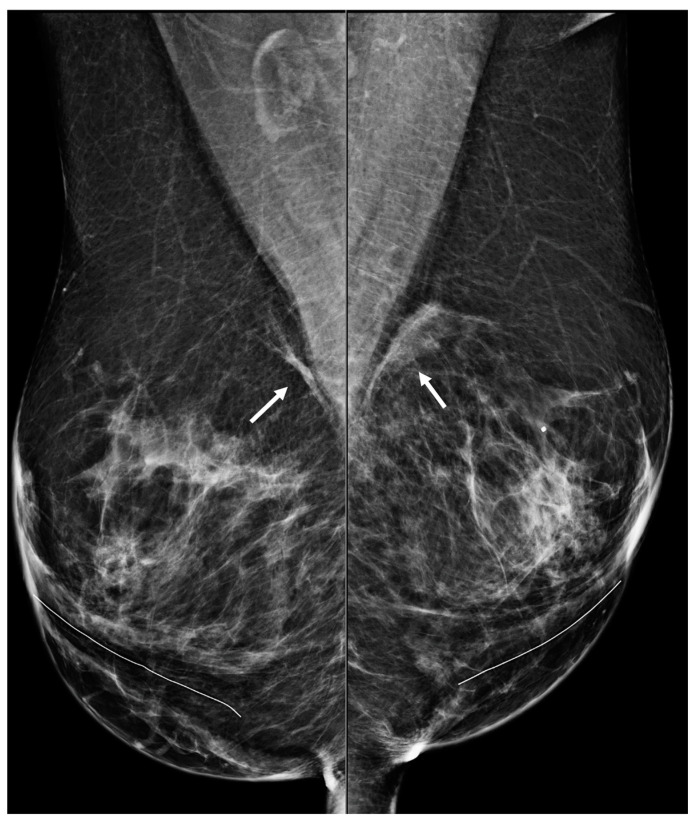
A 50-year-old woman with a history of subglandular silicone implants. Mammography with bilateral MLO views one year after BES demonstrates bilateral linear asymmetries (arrows) in the subglandular region.

**Figure 8:**
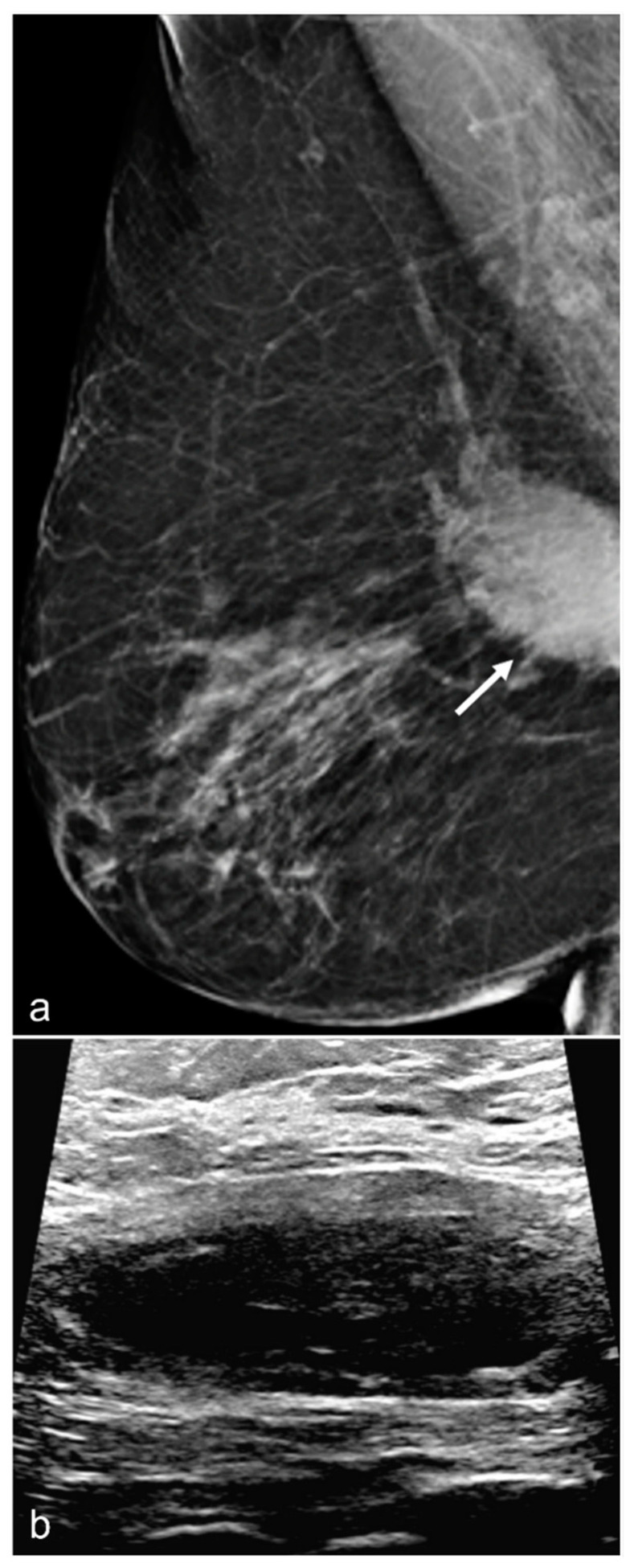
A 67-year-old woman with subglandular silicone implants developed a large, dense right subglandular mass (arrow) on mammography (**a**) two years after BES. Follow-up longitudinal right breast ultrasound (**b**) demonstrates a fluid collection with internal debris, confirming evidence of a seroma.

**Figure 9:**
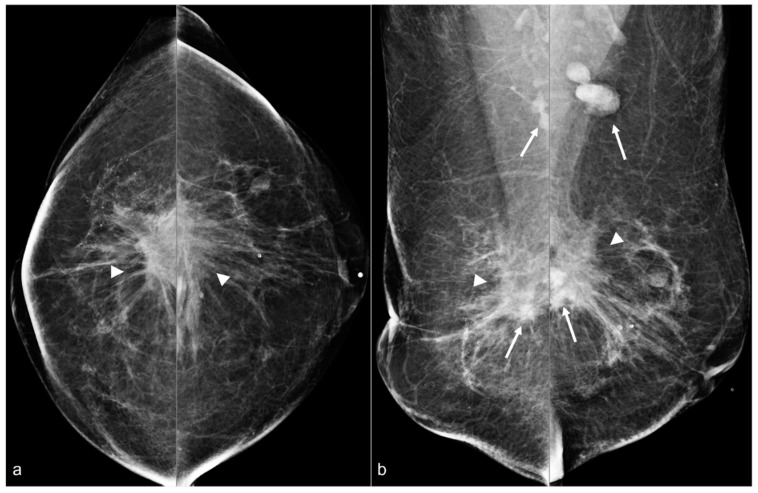
A 64-year-old woman status post BES due to silicone implant rupture. Spiculated masses (arrowheads) are seen in the pre-pectoral region on bilateral CC (**a**) and MLO (**b**) views. Hyperdense material (arrows) in both surgical beds and the bilateral axillae are sequelae of prior silicone implant rupture.

**Figure 10:**
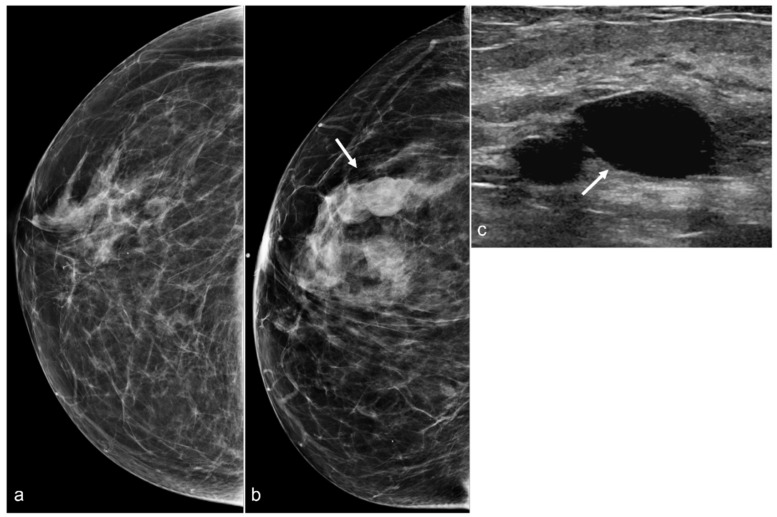
A 58-year-old woman with a history of BIA-ALCL and subsequent BES of retropectoral silicone implants. Post-BES CC mammogram (**a**) and most recent mammogram CC view (**b**) demonstrate developing masses (arrow). Right breast ultrasound in the transverse view (**c**) shows cysts (arrow).

Asymmetries and masses anterior to the pectoralis major muscle are common findings following BES. These findings are often seen in patients with a history of subglandular implants. Post-surgical findings with focal linear asymmetries are usually secondary to fibrosis (Figure 7). Hematomas and seromas may be seen on mammography as large dense masses (Figure 8). Ultrasound is helpful in showing the typical features of hematomas and seromas (Figure 9). Developing masses in patients who are status post BES should be carefully evaluated with mammography and ultrasound. Figure 10 shows developing masses in the region of the BES scar. A subsequent ultrasound showed cysts.

### 2.5. Implant Capsule/Calcifications

**Figure 11:**
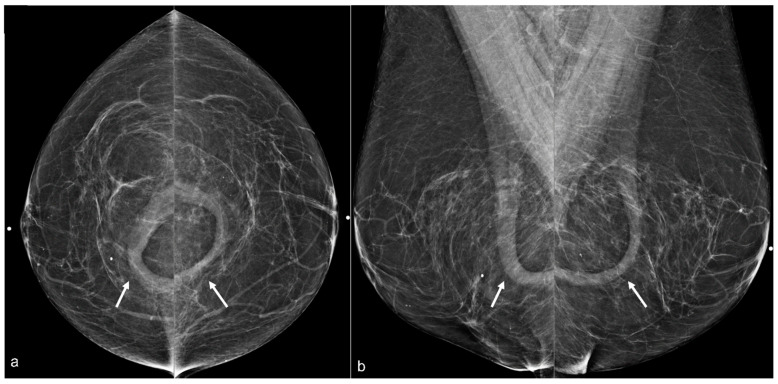
A 67-year-old woman with a history of subglandular silicone implants. Mammograms with bilateral CC (**a**) and MLO (**b**) views demonstrate curvilinear densities (arrows), consistent with residual implant capsules.

**Figure 12:**
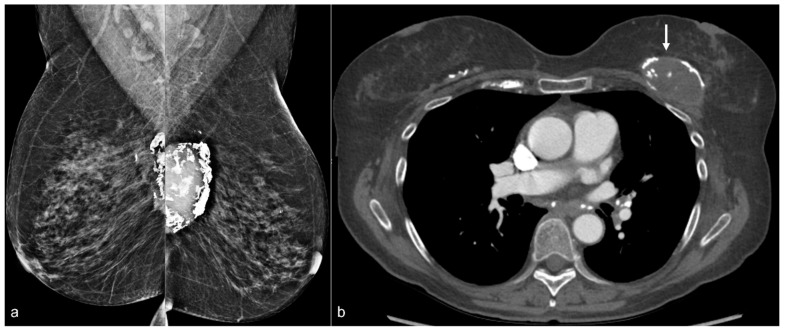
A 70-year-old patient’s mammogram (**a**) demonstrates bilateral calcified implant capsules with a dense mass in the left prepectoral location on the MLO view. CT (**b**) confirms a left breast seroma (arrow) with fibrous capsular calcifications.

In some cases, it is possible to see a residual implant capsule (Figure 11). The capsule may calcify in the expected prepectoral location (Figure 12).

### 2.6. Silicone Granulomas/Free Silicone

**Figure 13:**
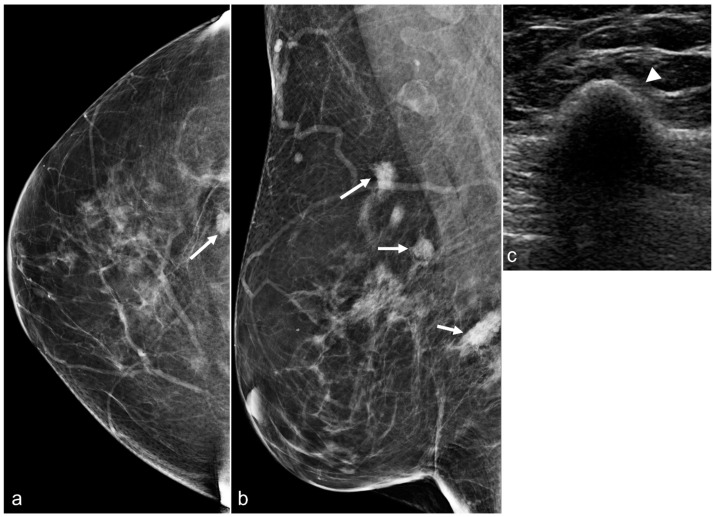
Mammograms of a 67-year-old woman with a history of retropectoral silicone implants and BES demonstrating several irregular dense breast masses (arrows) in the right breast on the CC (**a**) and the MLO (**b**) views. An ultrasound in the transverse view (**c**) confirms the classic snowstorm appearance of free silicone (arrowhead).

Rupture of a silicone implant with resultant free silicone/silicone granulomas in the breast and the axillary lymph nodes is a well-known complication. Silicone granulomas may present as masses (Figure 13).

### 2.7. Suspicious Findings

**Figure 14:**
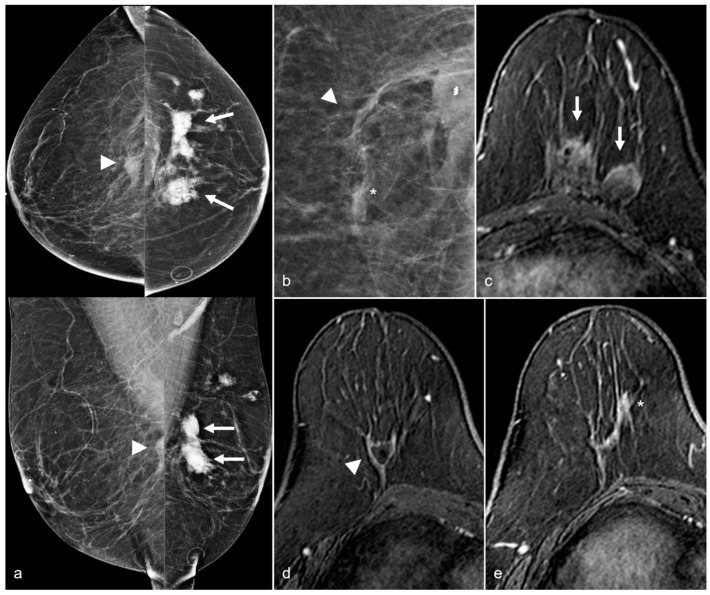
A 65-year-old woman with a history of a ruptured subglandular silicone implant. Screening mammogram (**a**) demonstrates very dense masses (arrows) in the left prepectoral region on the CC and the MLO views. Suspicious right breast calcifications (arrowhead) were biopsied with a biopsy clip seen in a hematoma in the right prepectoral region on the CC and MLO views. Pathology revealed ductal carcinoma in-situ. An additional biopsy was performed in the right breast for a small focal asymmetry (*) better seen on magnified CC view (**b**) and pathology demonstrated invasive ductal carcinoma. MRI demonstrates a T1 isointense peripherally enhancing silicone granuloma in the left breast prepectoral region ((**c**), arrows). In the right breast, a biopsy clip is seen within the biopsy cavity ((**d**), arrowhead). Anterior and inferior to this region (**e**), there is a linear non-mass enhancement. More anterior and medial to the non-mass enhancement, there is an enhancing mass (*), corresponding to the biopsy-proven invasive ductal carcinoma.

**Figure 15:**
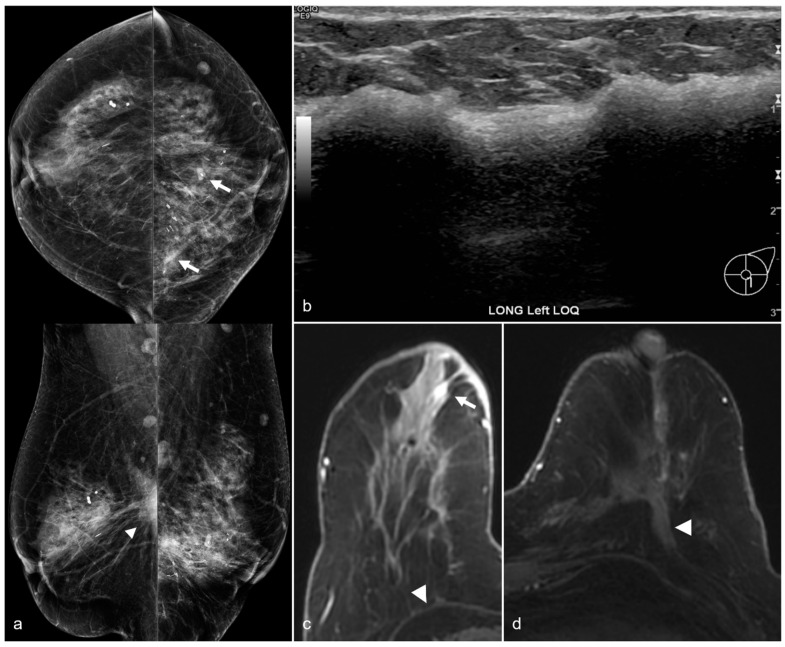
A 79-year-old woman with a history of subglandular silicone implants with a remote history of BES. Mammography (**a**) demonstrates dense masses in the left breast (arrows) and a right prepectoral asymmetry (arrowhead). Four biopsy clips are also seen in the left breast secondary to prior benign biopsies. Longitudinal left breast ultrasound (**b**) demonstrates an extensive snowstorm appearance, which limits evaluation of the breasts. T1-weighted post-contrast axial MRI (**c**) reveals an enhancing 0.3 cm focus in the right central breast (arrow). Subsequent MRI-guided biopsy revealed multifocal LCIS and ALH, and the patient underwent breast conservation therapy. Note the post-BES changes in the bilateral prepectoral regions (arrowheads), which is more pronounced in the left breast (**d**).

**Figure 16:**
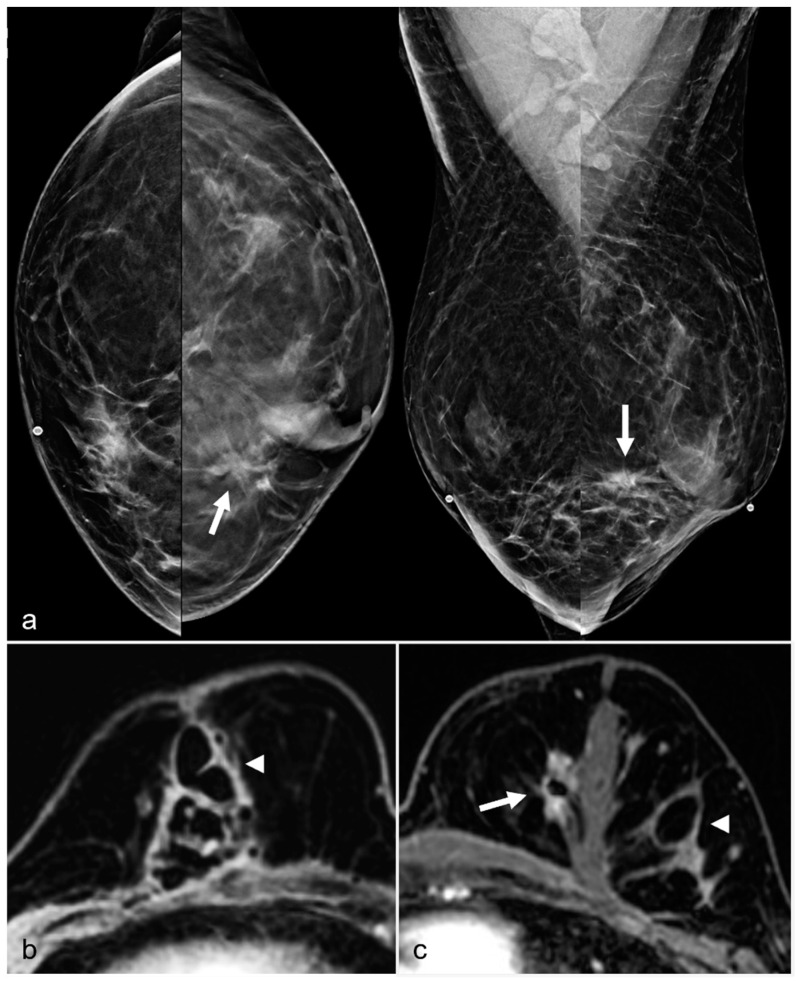
A 52-year-old woman with a history of subglandular silicone implants. Prior mammograms with implant displaced views were negative. A screening mammogram with bilateral CC and MLO views one year after BES (**a**) demonstrates a spiculated mass in the left lower inner quadrant (arrow), which was biopsied under ultrasound guidance with pathology revealing invasive ductal carcinoma. T1-weighted post-contrast MRI axial images demonstrate areas of fat necrosis in both breasts ((**b**,**c**), arrowheads). An enhancing mass with a biopsy clip is seen adjacent to the surgical scar ((**c**), arrow) in the left breast representing the biopsy proven carcinoma.

**Figure 17:**
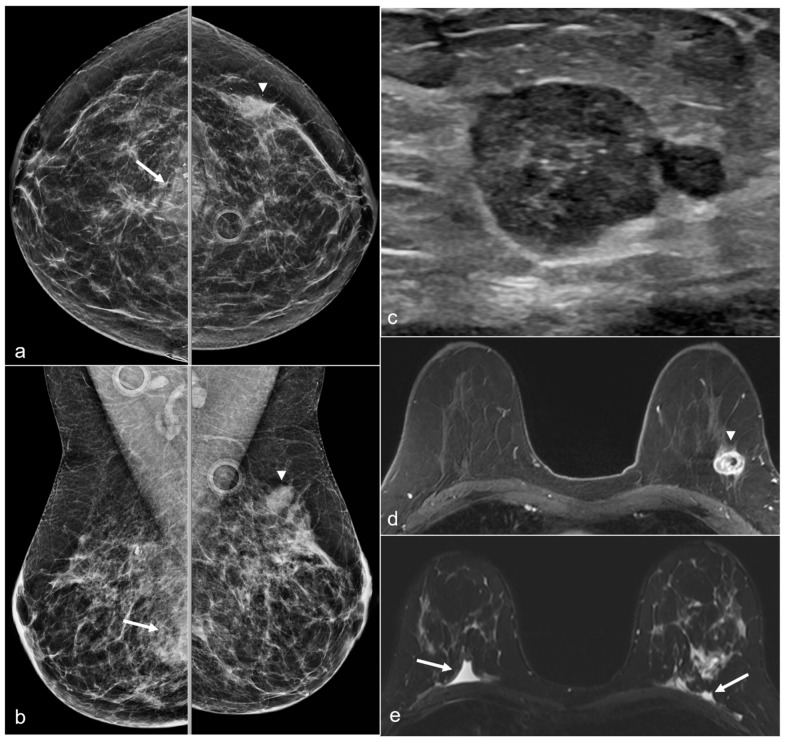
A 50-year-old woman with a history of subglandular silicone implants, status post BES. Screening mammograms with bilateral CC (**a**) and MLO views (**b**) demonstrate a mass in the left breast upper outer quadrant (arrowhead). BES changes are more prominent in the right breast with focal asymmetries in the prepectoral region (arrows) with dystrophic calcifications. Subsequent ultrasound examination of the left breast in the transverse plane (**c**) with ultrasound-guided biopsy revealed triple-negative invasive ductal carcinoma. T1-weighted post-contrast MRI (**d**) revealed a left breast enhancing mass (arrowhead) with a biopsy clip. There was also right greater than left prepectoral fluid (arrows) on the axial T2-weighted images (**e**).

Suspicious findings in patients with a history of BES need to be thoroughly evaluated. Comparison examinations can be helpful. Silicone granulomas may distract the radiologist’s attention away from findings suspicious for breast cancer (Figure 14). Silicone granulomas may obscure important findings (Figure 15). Developing masses are suspicious and require further evaluation (Figure 16 and Figure 17).

## 3. Discussion

Imaging findings following BES may differ according to the surgical procedure and the technique. Clinical history and prior imaging play an essential role in interpreting the imaging findings. Breast imagers should be aware of the expected findings following BES in order to prevent physical and emotional harm to patients by doing unnecessary follow-ups and/or biopsies.

An awareness of the anticipated findings following BES enables radiologists to discern findings suspicious for breast cancer that require additional evaluation and/or biopsy.

## Data Availability

No new data were created or analyzed in this study. Data sharing is not applicable to this article.
